# Participatory Development and Psychometric Evaluation of the Introspective Predictive Processing Inventory: A Self-Report Measure for Autistic and Non-Autistic Adults

**DOI:** 10.1177/13623613261443728

**Published:** 2026-05-03

**Authors:** Marik Roos, Hannah Storm, Lucie Zimmer, Tobias Schuwerk

**Affiliations:** 1 Universität Wien, Austria; 2 Ludwig-Maximilians-Universität München, Germany

**Keywords:** autism, predictive processing, self-report measure, psychometric validation, participatory research

## Abstract

Traditional self-report autism measures are often constructed from an “outside view” by non-autistic researchers rather than reflecting authentic autistic experiences. Predictive processing theory offers a framework for understanding autism, but comprehensive tools assessing the subjective manifestations of predictive processing differences and associated challenges have been lacking. This study aimed to develop and validate the Introspective Predictive Processing Inventory (IPPI), a self-report measure assessing predictive processing characteristics and their subjective consequences in everyday life. Through community-led, participatory research, we developed an initial 65-item version in German and English, and employed a five-stage validation approach across three samples (*N* = 790). We used network-based item optimization, exploratory and confirmatory factor analyses, measurement invariance testing, and convergent validity assessment. Network optimization reduced the scale to 18 items while maintaining excellent reliability and improved discriminative power. Exploratory factor analysis revealed a stable two-factor structure: “Prediction Integration and Interpretation” and “Prediction Error Sensitivity and Stability Needs”. The IPPI demonstrated exceptional discriminative validity (area under the curve >0.97), strong convergent validity with established measures, measurement invariance across groups, and independence from general cognitive abilities. It provides a tool for assessing predictive processing experiences and their daily consequences, advancing autism research that bridges predictive processing theory with lived experiences.

**Lay Abstract:**

Most questionnaires used to understand autism are created by non-autistic researchers who imagine what autism might be like, rather than capturing what autistic people actually experience. Scientists have a theory called “predictive processing” that suggests our brains are constantly trying to predict what will happen next in our environment. When these predictions don’t match reality, it can cause stress and difficulties in daily life. However, there was no good way to measure these internal experiences and daily challenges that autistic people face. To address this gap, an autistic researcher worked with autistic community members and non-autistic researchers to create a questionnaire called the Introspective Predictive Processing Inventory (IPPI). They started with 65 questions, developed both German and English versions, and tested it with 790 autistic and non-autistic adults from mostly Germany and the United Kingdom. Using advanced statistical methods, they refined it down to 18 key questions that capture two main areas: difficulties understanding and integrating information from social situations and the environment, and sensitivity to unexpected changes with strong needs for predictability, causing distress when things don’t go as expected. The final 18-question IPPI was highly reliable and could accurately distinguish between autistic and non-autistic people 97% of the time. Importantly, these differences were not related to intelligence levels. These findings provide researchers and clinicians with a new tool to understand the internal experiences of autistic people from their own perspective. This could help develop better support strategies, improve quality of life, and advance autism research that truly reflects autistic experiences rather than outside assumptions about autism.

## Introduction

Autism research faces an important measurement challenge: Traditional self-report assessment tools are often constructed from an “outside view”—items phrased by non-autistic researchers imagining autistic experience rather than capturing authentic internal experiences. In addition, these measures are typically grounded in diagnostic symptom criteria rather than in theoretical frameworks that explain underlying cognitive mechanisms. Widely used instruments like the Autism Quotient (AQ; Baron-Cohen et al., 2001) exemplify both limitations. There is a growing need for theory-based instruments developed in partnership with autistic people that illuminate autistic internal experiences and link cognitive processes to symptomatology.

The predictive processing theory offers a promising framework for understanding autism by focusing on internal cognitive mechanisms that may manifest in observable experiences and challenges. According to this theory, the brain continuously generates predictions about incoming sensory information and updates these models when predictions fail (“prediction errors”; Clark, 2013). This involves a hierarchical system where higher-level brain areas send predictions downward while lower levels send prediction errors upward, with precision weighting determining their influence on perception and behavior. These predictions are based on prior knowledge (“priors”) from past experience. The theory has been extended to interpersonal communication, where successful social interaction depends on mutual predictive modeling (Friston & Frith, 2015; Koster-Hale & Saxe, 2013).

Several theoretical accounts propose that autism involves differences in predictive processing mechanisms. Pellicano and Burr (2012) hypothesized that autistic people have reduced influence of prior expectations on perception (“hypo-priors”). Following this hypothesis, their reliance on the actual sensory input seems to be higher, resulting in a less experience-modulated, but more veridical sensory perception, which could explain various sensory characteristics in autism. Furthermore, by relying less on cognitive predictions, autistic individuals may experience the social world as more unpredictable than non-autistic individuals, which can lead to stressful and overwhelming experiences in social interaction (Bolis et al., 2017). At the same time, Van de Cruys et al. (2014) proposed that autistic people tend to expect a high precision between predicted and actual experiences, reflecting overly precise priors. Consequently, prediction errors tend to be overweighted, processed inflexibly, and poorly integrated, while priors resist updating, leading to repetitive behaviors as an attempt of a successful and reassuring predictive experience and an increased need for environmental predictability. Sinha et al. (2014) emphasized disrupted temporal prediction, where difficulties predicting when events occur contribute to timing and sequential processing challenges. While integration into a unified framework remains unclear, these accounts may complement each other by explaining different predictive processing domains.

The relationship between these theoretical accounts remains an active area of investigation. Rather than representing a single unified mechanism, these frameworks may describe different aspects of predictive processing that are differentially affected in autism (Angeletos Chrysaitis & Seriès, 2023). Hypo-priors and precision weighting differences may characterize how predictions are formed and weighted relative to sensory input, while differences in prediction error processing may characterize responses when predictions are violated. In addition, these mechanisms may operate differently across domains, for instance, affecting the learning of complex social expectations more than simple perceptual regularities.

Empirical support has grown substantially, with reviews indicating differences in sensory prediction, temporal processing, and statistical adaptation (Angeletos Chrysaitis & Seriès, 2023; Cannon et al., 2021). However, evidence remains mixed, suggesting the relationship is more nuanced than initially proposed. Albeit this mixed evidence, predictive processing theories offer a cognitive framework capable of accounting for a broad range of autistic characteristics within a unified mechanism, spanning sensory processing differences, repetitive behaviors, need for sameness, and social communication characteristics.

These predictive processing differences manifest in observable daily experiences. For instance, when precision weighting assigns excessive weight to bottom-up sensory inputs, individuals may experience overwhelming sensory environments (Pellicano & Burr, 2012). When communication partners operate with divergent generative models, mutual understanding fails, leading to experiences of being misunderstood or misinterpreted (Friston & Frith, 2015). When prediction errors are processed inflexibly, unexpected changes cause disproportionate distress (Van de Cruys et al., 2014). A measure capturing these subjective manifestations of predictive processing differences can bridge theoretical frameworks with lived experience.

Despite predictive processing’s theoretical promise, until recently, there was no comprehensive self-report tool to assess predictive processing characteristics in daily life. This absence limited researchers’ ability to connect theoretical frameworks with individuals’ lived experiences and functional outcomes. A significant advance came with O’Brien et al. (2025), who developed the Prediction-Related Experiences Questionnaire (PRE-Q), a 19-item self-report measure of prediction skills across sensory, motor, and social domains. The PRE-Q items assess concrete prediction abilities such as catching a ball, anticipating food texture from appearance, or predicting what someone will say next.

However, important gaps remain. The PRE-Q focuses primarily on prediction accuracy and behavioral outcomes rather than the underlying cognitive processes and their subjective consequences, including the daily challenges that arise from differences in predictive processing mechanisms in autism. The PRE-Q development, like other recent examples (Hull et al., 2019), notably incorporated input from autistic individuals, but the broader landscape of participatory-developed autism measures remains limited. Furthermore, emerging evidence suggests that differences in predictive processing may contribute to anxiety and other mental health challenges commonly experienced by autistic individuals (Stark et al., 2021), highlighting the clinical relevance of better understanding these internal processes. These examples reflect a growing recognition that measures designed to capture autistic experiences and serve as practical clinical tools must be developed in partnership with autistic people.

Given this theoretical complexity, what is needed is a comprehensive measure capturing the internal experiences and daily consequences that predictive processing theories propose as central to autism. Rather than targeting a single theoretical mechanism, such a measure should assess experiences that could arise from multiple predictive processing differences, including both how predictions are formed and how violations of those predictions are processed and responded to in daily life. Such a tool would provide deeper insights into how differences in predictive processing manifest in everyday life and their impact on well-being, functioning, and clinical outcomes. It would advance the field in three key ways. From a research perspective, it would enable testing specific theoretical predictions (e.g., whether hypo-priors predict sensory sensitivities, whether prediction error sensitivity relates to anxiety) and examining relationships between self-reported predictive processing experiences and objective measures like neural prediction error responses. Clinically, it would offer an efficient assessment tool for understanding individual differences in predictive processing characteristics, which could inform targeted supports, such as environmental modifications to increase predictability, interventions addressing anxiety related to prediction error sensitivity, or communication strategies accounting for divergent predictive models. More broadly, it would facilitate translation of theoretical frameworks into empirical research by providing a practical measurement approach.

The present study aimed to develop and psychometrically evaluate the Introspective Predictive Processing Inventory (IPPI), a comprehensive self-report measure designed to assess predictive processing characteristics and their daily consequences in autistic and non-autistic adults. This work was conducted by autistic and non-autistic researchers in participatory collaboration. Beginning with an initial 65-item version, we employed a comprehensive five-stage validation approach across three distinct samples: (1) network-based item selection and exploratory factor analysis (EFA) in a matched sample of autistic and non-autistic adults, (2) factor structure analysis and cross-group measurement invariance testing, (3) discriminative and convergent validity assessment, (4) confirmatory factor analysis in an independent autistic sample, and (5) extended validation in a laboratory-based sample with cognitive ability controls.

## Method

We report how we determined the sample size, all data exclusions, all manipulations, and all measures in this study. Material, data, and analysis scripts of this study can be found at Open Science Framework (OSF; https://osf.io/uftvx/). An interactive version of the validated 18-item IPPI is freely accessible at https://ippi-tool.netlify.app/. The tool runs entirely in the user’s browser and does not collect or store any data. Demographic details of participants are not shared as it cannot be guaranteed that it is impossible to identify individual data sets.

### Participatory Methods

This study represents a community-led participatory research approach. The first author (M.R.), who is autistic, developed the initial 65-item IPPI version and conducted initial pre-testing in an anonymous online study. Feedback from autistic participants during this pilot phase was incorporated into the questionnaire development in both German and English versions. The non-autistic co-authors (T.S., H.S., L.Z.) provided the institutional framework and resources to conduct the validation study. Throughout the research process, all authors collaboratively discussed item wording, factor structure interpretation, and theoretical implications. Following the hierarchy of participatory research as described in den Houting et al. (2021), this project can be classified as “community-led,” representing “doing with” and “getting help we asked for” approaches to autism research. This participatory approach shaped item development in several ways. Rather than focusing solely on observable behaviors, items capture internal experiences and consequences that autistic individuals report but may not be visible to external observers. For example, items assess the effort required for social camouflaging (IP38: “It takes a lot of strength for me to appear ‘normal’ to others”), delayed interoceptive awareness (IP50), and recovery time from everyday challenges (IP60). In addition, items addressing communication difficulties frame these as bidirectional rather than assuming unidirectional deficits (IP10, IP18), reflecting a perspective that emerged from autistic self-advocacy (Milton, 2012).

### Participants

A total of 473 autistic (*M_age_* = 38.76 years, *SD_age_* = 12.28 years, range: 18–72, 268 female, 144 male, 61 non-binary) and 317 non-autistic adults (*M_age_* = 29.63 years, *SD_age_* = 11.13 years, range: 18–66, 225 female, 85 male, 7 non-binary) took part in the study. Additional 279 participants from the Autism group were excluded from the final sample because they did not complete the full survey (*n* = 259), were under 18 years old (*n* = 3), did not provide diagnosis details (*n* = 5), were duplicate data sets (*n* = 6), or were identified as “straightliner” (i.e., clicking the same value for all items) in one or more of the used questionnaires (*n* = 6). From the Comparison group, a total of 79 additionally tested participants were excluded because they did not complete the full survey (*n* = 77), were under 18 years old (*n* = 1), or were identified as straightliner (*n* = 1).

Participants were recruited via convenience sampling through social media, personal networks, autism organizations, and online communities. Most participants were from Germany, with a U.K. subsample for English validation (see Supplemental Material, Table S1). Participants were ≥18 years old. Autistic participants either had a formal autism diagnosis or self-identified as autistic. A subsample of participants with a formal diagnosis verified their diagnosis by providing medical records or an equivalent document stating details about their diagnostic status (see Procedure for details). Preliminary analyses showed no IPPI score differences between formally diagnosed, verified, and self-identifying participants, so groups were combined, as well as between German and English versions in either group (see Supplemental Material S2).

Dropout rates differed between groups: 34.4% in the autism group (259 of 752 who initiated) versus 19.4% in the comparison group (77 of 396). The higher attrition in the autism group likely reflects recruitment where survey links were publicly shared. Given the study’s focus on autism and autistic experiences, autistic individuals may have been motivated to explore the survey out of personal interest, even without firm intention to complete it, whereas non-autistic individuals had less personal motivation to even access the survey. In addition, survey length (20–40 min) may have exceeded expectations for those initially exploring the content. The introspective nature of the questions, requiring careful reflection on internal experiences, may have contributed to longer completion times and greater cognitive fatigue.

Non-autistic participants from the Comparison group were only included if they had no suspected diagnosis of autism. Other psychiatric or neurological conditions were not exclusion criteria in order to avoid further bias through sample selection (cf. Schwartz & Susser, 2011). The sample size was determined based on cost-effectiveness considerations. We aimed to collect as much data as possible within the constraints of available financial and personnel resources. All participants provided informed written consent. Autistic participants who took part online and agreed to verify their autism diagnosis were able to choose to enter a random draw for a chance to get one of 20 Amazon gift cards with a value of 25€/17£/26$. Participants who participated in the lab received monetary compensation.

Due to our recruitment strategy, we were not able to anticipate the sample composition in terms of demographic characteristics or diagnoses. Therefore, we drew three subsamples from the overall participant pool (Table 1 provides an overview of descriptive statistics characterizing the samples; Supplemental Table S3 provides details on the percentages of further mental health diagnoses per sample).

**Table 1.** Demographic Information for Autistic and Non-autistic Participants, Presented Separately for Each Subsample. Results From Independent *t*-Tests and Effect Sizes Indicate Successful Group Matching.

	Autism group	Comparison group	Matching		
			*t* value	*p* value	effect size
Sample 1					
Size	*n* = 196	*n* = 196			
Age in years (*M* [*SD; range*])	34.85 (13.81; 18–72)	32.39 (12.22; 18–66)	*t*(384) = 1.86	*p* = .063	*d* = 0.19
Age at diagnosis (*M* [*SD; range*])	30.53 (14.57; 4–69)	–			
Gender (f, m, nb)	137, 52, 7	137, 52, 7			
Sample 2					
Size	*n* = 247	–			
Age in years (*M* [*SD; range*])	42.63 (9.61; 18–66)	–			
Age at diagnosis (*M* [*SD; range*])	38.53 (10.70; 2–66)	–			
Gender (f, m, nb)	115, 80, 52	–			
Sample 3					
Size	*n* = 30	*n* = 30			
Age in years (*M* [*SD; range*])	32.43 (10.66; 18–52)	33.73 (9.69; 21–59)	*t*(57) = –0.49	*p* = .623	*d* = –0.13
Age at diagnosis (*M* [*SD; range*])	27.20 (12.22; 5–52)	–			
Gender (f, m, nb)	16, 12, 2	18, 12, 0			
Verbal IQ^a^ (*M* [*SD; range*])	111 (15; 92–143)	116 (18; 88–145)	*t*(56) = –1.08	*p* = .284	*d* = –0.28
Nonverbal IQ^b^ (*M* [*SD; range*])	111 (13; 86–138)	117 (9; 98–137)	*t*(50) = –1.81	*p* = .076	*d* = –0.47

*Note. M* = mean, *SD* = standard deviation, f = female, m = male, nb = non-binary.

a MWT-B = Mehrfachwahl-Wortschatztest (German multiple-choice vocabulary test). ^b^ CFT-20-R = Culture-Fair Test 20-R (fluid intelligence test).

#### Sample 1

From the overall participant pool, we created matched subsamples using nearest-neighbor propensity score matching for age and gender, resulting in Sample 1 (196 autistic, 196 non-autistic participants; see Supplemental Material S4 for detailed procedures). This sample was used for the initial item analysis, EFA, and measurement invariance testing. In accordance with International Classification of Diseases, 10th Revision (ICD-10) criteria, autistic participants reported the following diagnosis types: Childhood Autism (F84.0, *n* = 4), Atypical Autism (F84.1, *n* = 2), Asperger’s Syndrome (F84.5, *n* = 122), and Pervasive Developmental Disorder, unspecified (F84.9, *n* = 39). A total of 11 additional autistic participants labeled themselves as “high-functioning,” a term that remains relatively common in Germany. A total of 12 participants described their (self-identified) autism diagnosis in a free-text response. Another six participants did not specify their diagnosis type. Of the 196 autistic participants, 38 (19.39%) were self-identifying autistic individuals without a formal diagnosis, while the remaining participants held a formal diagnosis as described above.

#### Sample 2

Sample 2 consisted of the remaining 247 autistic participants who were excluded from the matching procedure used to create Sample 1. As a result, this sample was more heterogeneous in its demographic characteristics and differed in these from Sample 1. We leveraged this variation to conduct a confirmatory factor analysis, aiming to test whether the factor structure identified in the EFA of Sample 1 could be replicated and thus generalized to an independent sample with different demographic properties, especially regarding age and gender. Note that 91 non-autistic participants who were left over after the matching procedure were not included in further analyses. In Sample 2, autistic participants reported the following diagnosis types: Childhood Autism (F84.0, *n* = 3), Atypical Autism (F84.1, *n* = 7), Asperger’s Syndrome (F84.5, *n* = 127), and Pervasive Developmental Disorder, unspecified (F84.9, *n* = 59). A total of 20 additional autistic participants labeled themselves as “high-functioning,” and 19 participants described their (self-identified) autism diagnosis in a free-text response. Additional 12 participants did not specify their diagnosis type. Of the 247 autistic participants in Sample 2, a total of 51 (20.65%) were self-identifying autistic individuals, while the remaining participants held a formal diagnosis as described above.

#### Sample 3

Data from Sample 3 stem from 30 autistic and 30 non-autistic participants who came to the lab for an independent fMRI study (Zimmer et al., 2025). These participants were recruited independently and did not participate in the online data collection for Samples 1 or 2, ensuring complete independence across validation samples. The two groups were matched by age, gender, and verbal intelligence (a German multiple-choice vocabulary test; Mehrfachwahl-Wortschatz-Intelligenztest, MWT-B; Lehrl, 2005) and nonverbal intelligence (Culture-Fair Test 20-R; CFT-20-R; Weiß, 2006). In this sample, autistic participants indicated the following diagnosis types: Atypical Autism (F84.1, *n* = 1), Asperger’s Syndrome (F84.5, *n* = 23), and multiple autism diagnoses (*n* = 3). An additional two autistic individuals described themselves as “high-functioning,” and one did not specify their exact diagnosis type.

### Procedure

For all samples, data were collected online using SoSci Survey (Leiner, 2019). Participants received study information, provided informed consent, and then completed demographics and questionnaires (IPPI, AQ, Broad Autism Phenotype Questionnaire [BAP-Q]) in fixed order for comparability. The study took 20 to 40 min, was anonymous, and available in German or English. Following the initial online questionnaire data collection, Sample 3 participants completed an in-person laboratory study. They participated in an fMRI study (Zimmer et al., 2025) and then completed intelligence tests. Data collection occurred between May 2022 and June 2023.

### Materials

#### Initial 65-Item Version of Introspective Predictive Processing Inventory

The initial IPPI version (Roos, 2020) is a 65-item self-report questionnaire assessing predictive processing characteristics and their daily consequences in autistic and non-autistic adults. Item development was informed by conversations with autistic individuals and predictive processing literature (e.g., Holroyd & Coles, 2002; Pellicano & Burr, 2012; Van de Cruys et al., 2014). Items were designed to capture the subjective manifestations of predictive processing differences in daily life. Rather than assessing prediction accuracy directly, items focus on how differences in predictive mechanisms manifest as observable experiences and challenges. For example, items assess experiences arising from precision weighting imbalances (e.g., sensory overwhelm from excessive bottom-up precision), prediction error sensitivity (e.g., distress from unexpected events), difficulties with predictive integration (e.g., uncertainty in interpreting social cues), and challenges with predictive model updating (e.g., difficulty adapting internal models). This approach enables assessment of the daily consequences of predictive processing differences as experienced by autistic individuals. Items were not developed for specific subscales; factor structure was determined empirically. Following a pilot study (*n* = 314) that removed 15 poorly discriminating items from 80 original items and revised others for clarity, the current 65-item version was finalized (Supplemental Material S5 and S6). Respondents use 7-point scales (1 = “do not agree at all/very atypical” to 7 = “agree very strongly/very typical”). Total scores are summed across items, with four items reverse-coded (6, 13, 21, and 55).

#### Autism Quotient

The Autism Spectrum Quotient (AQ) assesses autistic traits in adults and adolescents (Baron-Cohen et al., 2001; German version: Freitag et al., 2007). The 50-item questionnaire spans five subscales (Social Skill, Attention Switching, Attention to Detail, Communication, Imagination) rated on 4-point scales. Responses are dichotomized and summed (range: 0–50), with scores ≥32 indicating clinically relevant autistic traits (sensitivity = .79, specificity = .98). Internal consistency ranges from α = .63 to α = .77.

#### Broad Autism Phenotype Questionnaire

The BAP-Q assesses autism-related traits in non-autistic individuals (Hurley et al., 2007). The 36-item self-report measure uses 6-point scales across three subscales: Aloof Personality, Rigid Personality, and Pragmatic Language. Total scores range from 1 to 6, with higher scores indicating greater trait expression. Internal consistency is high (α = .95 total scale, α = .85–.94 subscales). A cutoff of 3.15 provides optimal sensitivity and specificity (both approaching .80). A German version of the questionnaire is available (Schuwerk et al., 2019).

### Analyses

Data preprocessing and analyses were performed using R version 4.4.2 (R Core Team, 2024) and R studio version 2024.12.0+467 (Posit Team, 2024). After data import, preprocessing, preliminary analyses, and participant matching, we employed a comprehensive five-stage validation approach designed to establish the IPPI’s psychometric properties through progressively independent samples:

#### Step 1: Network-Based Item Selection and Optimization of the IPPI in Sample 1

In Sample 1, in which autistic and non-autistic participants were matched for age and gender, we explored their performance in the IPPI and the other questionnaires. Subsequently, we performed an item analysis to identify an optimal subset of items from the initial 65-item version of the IPPI. We implemented a multi-strategy approach combining network analysis and psychometric properties for item selection. Item-level Cohen’s *d* values assessed discriminative power, while a network was constructed from items with inter-correlations > 0.70. Community structures were identified using the walktrap algorithm (Pons & Latapy, 2005), and centrality measures (degree and eigenvector centrality) were calculated. Four complementary selection strategies balanced network coverage with discriminative power: (1) community representation, (2) isolated discriminators, (3) central hub items, and (4) top discriminators. The final optimized item set comprised unique items from these strategies, maintaining psychometric quality and construct coverage while minimizing redundancy (see Supplemental Material S7 for detailed methodological framework and selection criteria).

#### Step 2: Factor Structure Analysis and Cross-Group Measurement Invariance in Sample 1

EFAs using the *psych* package (Revelle, 2024) were conducted to identify underlying factor structures in the optimized IPPI item set. Analyses were performed on the total Sample 1 and separately for the Autism and Comparison groups to examine potential differences in factor structure. Parallel analysis using Principal Axis Factoring (PAF) was employed to determine the optimal number of factors, as recommended for ordinal/Likert-type-scale data (Costello & Osborne, 2005). An oblimin rotation was applied to allow for correlated factors. Prior to analysis, data factorability was assessed using Bartlett’s test of sphericity and Kaiser–Meyer–Olkin (KMO) measure. Following the EFA, we tested measurement invariance across the Autism and Comparison groups using the *lavaan* package (Rosseel, 2012). We examined configural, metric, and scalar invariance sequentially to assess whether the instrument demonstrated equivalent factor structure, factor loadings, and item intercepts across groups. We conducted EFAs on both the combined sample and separately by group to determine whether between-group differences might obscure within-group factor structure, with the autism group structure serving as the baseline for MI testing given that autistic individuals are the primary target population.

#### Step 3: Discriminative and Convergent Validity of Optimized Item Set Using Sample 1

We evaluated the effectiveness of the optimized item set in differentiating between the Autism and the Comparison group using an independent *t*-test. The discriminative validity of the optimized scale was evaluated through receiver operating characteristic (ROC) analysis using the *pROC* package (Robin et al., 2011). The optimal classification threshold was determined using Youden’s index (maximizing sensitivity + specificity − 1). At this threshold, we calculated sensitivity, specificity, and overall classification accuracy. The area under the curve (AUC) quantified the scale’s overall discriminative performance. Gender differences were examined using a linear regression model. We finally assessed the convergent validity of the optimized IPPI scale by examining its relationships with established autistic trait measures. Pearson correlations were calculated between the optimized IPPI sum score, AQ, and BAP-Q.

#### Step 4: Confirmatory Factor Analysis in Sample 2

To validate the factor structure identified through EFA, we conducted confirmatory factor analysis (CFA) in the independent Sample 2 using the *lavaan* package. Given the ordinal nature of the Likert-type-scale responses, we employed the robust weighted least squares mean and variance adjusted (WLSMV) estimator. Model fit was evaluated using standard indices including comparative fit index (CFI), Tucker–Lewis index (TLI), root mean square error of approximation (RMSEA), and standardized root mean square residual (SRMR). Internal consistency reliability was assessed using McDonald’s omega coefficients calculated with the semTools package (Jorgensen et al., 2021). We examined whether the factor structure maintained stability across samples with different demographic characteristics, focusing on factor loadings, correlations, and overall model fit.

#### Step 5: Extended Validation and Utility Assessment in Sample 3

We conducted a validation of the optimized IPPI in the independent Sample 3 to assess its discriminative power, convergent validity, and independence from cognitive abilities. The discriminative ability between the Autism and the Comparison group was evaluated using *t*-tests and Cohen’s *d* effect size calculations. To assess discriminative validity in Sample 3, we replicated the ROC analysis described in Step 3, calculating classification accuracy and sensitivity/specificity at the optimal threshold (Youden’s index). Convergent validity was likewise reassessed by correlating the IPPI with AQ and BAP-Q scores within each group, to confirm the robustness of prior associations in an independent sample. To assess the independence of our measure from general cognitive abilities, we performed multiple regression analysis with IPPI score as the dependent variable and group, verbal and nonverbal IQ as predictors to control for potential confounding effects of intelligence. Finally, we validated the factor structure identified in previous steps using confirmatory factor analysis.

## Results

### Step 1: Network-Based Item Selection and Optimization of the IPPI in Sample 1

An initial analysis of the full 65-item version revealed very high internal consistency (Cronbach’s α = .99). Item-level comparisons yielded Cohen’s *d* values ranging from 0.80 to 2.44, with all items showing significantly higher means in the Autism versus Comparison group (details in Supplemental Material S8).

**Figure 1.**
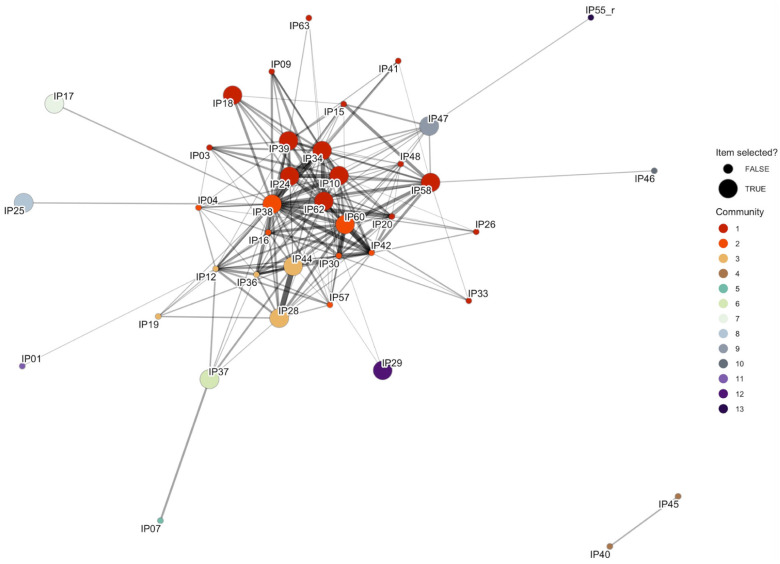
Network visualization of IPPI items with inter-correlations > 0.70 (39 of 65 items). Each node represents an item, with node color indicating community affiliation. Large nodes represent the 18 items selected for the final IPPI. Edges represent correlations between items, with edge thickness and transparency reflecting correlation strength (thicker and more opaque = stronger correlation). The 26 items not shown fell below the 0.70 threshold but remained selection candidates through discriminative power criteria.

For network visualization, we applied a correlation threshold of 0.70, resulting in 39 of the 65 items appearing in the network. Items below this threshold remained candidates for selection through discriminative power criteria. The resulting network revealed a densely interconnected central cluster of strongly correlated items, suggesting redundancy within this core item set (see Figure 1). Using the walktrap community detection algorithm, we identified 13 communities among the networked items, although many were quite small, consisting of only item pairs or single items (Community 1: 16 items, Community 2: seven items, Community 3: five items, Community 4: two items, Communities 5–13: one item each). Some items emerged as highly connected nodes, likely reflecting central facets of the underlying construct and thus playing a pivotal role in the questionnaire. In contrast, several peripheral and isolated items were identified, which may capture distinct or more specific aspects of the construct. The multi-strategy item selection process identified distinct yet overlapping sets of items. Several items were selected through multiple strategies, highlighting their multifaceted importance as community anchors, structural hubs, and powerful discriminators. The resulting set of selected items comprising the 18-item IPPI version can be found in Appendix 1 (optimized German version in Supplemental Material Table S9).

The final 18-item set (Appendix 1) demonstrated excellent reliability (α = .97), retaining 98.6% of original internal consistency with 22% improved discriminative power (average *d* = 1.94 vs. 1.59). The selection covered eight of 13 network communities.

### Step 2: Factor Structure Analysis and Cross-Group Measurement Invariance in Sample 1

Data met factor analysis assumptions (Bartlett’s test *p* < .001; KMO = 0.90–0.98). Parallel analysis revealed one factor for the combined sample but two factors when groups were analyzed separately. We conducted EFAs using PAF with oblimin rotation, extracting structures as indicated by parallel analysis. This approach allowed us to examine how factor structures differ when group-specific variance is isolated versus when between-group differences dominate in the combined sample. The one-factor combined solution likely reflects the dominance of between-group variance, while both groups showed substantively similar two-factor structures when analyzed separately.

For the full sample EFA with one factor, the model showed mixed fit indices (TLI = 0.93 indicating good fit, RMSEA = 0.09 indicating mediocre fit, 90% CI [0.08, 0.10]). All 18 items loaded strongly on the single factor, with standardized loadings ranging from 0.71 to 0.92. This general factor explained 68% of the total variance, suggesting a robust underlying construct. The highest loading items were IP38 (“It takes a lot of strength for me to appear ‘normal’ to others”; 0.92), IP34 (0.89), and IP24 (0.88). Factor score reliability was excellent (correlation with factor = 0.99, multiple *R*^2^ = .98).

**Figure 2.**
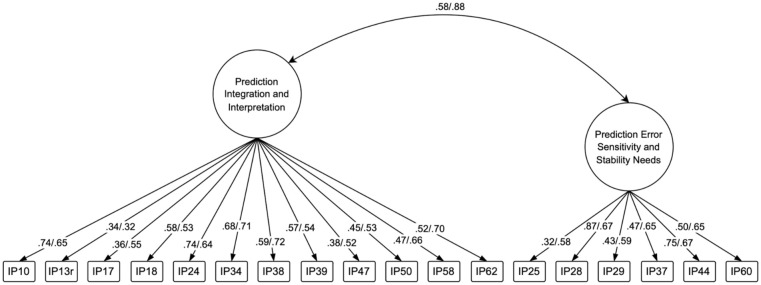
Two-factor model of the IPPI showing standardized factor loadings and interfactor correlation. The model displays 18 items loading onto two correlated factors: Prediction Integration and Interpretation (12 items) and Prediction Error Sensitivity and Stability Needs (six items). Standardized factor loadings are presented from the EFA in the Autism group of Sample 1 (on the left) and the CFA in Sample 2 (on the right). The interfactor correlation was *r* = .88. All factor loadings were statistically significant (*p* < .001).

For the Autism group, the two-factor EFA showed good fit indices (TLI = 0.92, RMSEA = 0.06, 90% CI [0.04, 0.07]). The two factors explained a modest 38% of the total variance (Factor 1: 24%, Factor 2: 15%). Factor 1 contained items related to prediction integration and interpretation, while Factor 2 captured prediction error sensitivity and stability needs (Figure 2; Table S10 in the Supplemental Material provides an overview of factors, items, and their conceptual relation to the construct). The factors were moderately correlated (*r* = .58), suggesting related but distinct constructs. Factor score reliability was very good for both factors (correlation with factors: 0.94 and 0.92, respectively).

For the Comparison group, the two-factor EFA showed acceptable to good fit indices (TLI = 0.90, RMSEA = 0.08, 90% CI [0.06, 0.09]). The two factors explained 50% of the total variance (Factor 1: 35%, Factor 2: 15%), which is notably higher than in the Autism group and meets the conventional threshold for adequate factor solutions. Factor 1 comprised items related to prediction integration and interpretation, with highest loadings from IP34 (0.87), IP24 (0.86), and IP10 (0.77). Factor 2 included items reflecting prediction error sensitivity and stability needs, with highest loadings from IP44 (0.80), IP28 (0.77), and IP37 (0.60). The factors were moderately correlated (*r* = .67), suggesting related aspects of the construct. Factor score reliability was very good for both factors (correlation with factors: 0.97 and 0.93, respectively). Despite differences in explained variance, both groups showed similar two-factor structures representing social-communicative challenges and need for predictability.

For measurement invariance testing, we specified a two-factor model based on the Autism group EFA results, as both groups revealed similar two-factor structures and autistic individuals represent the primary target population: “Prediction Integration and Interpretation” (12 items) and “Prediction Error Sensitivity and Stability Needs” (six items). Two error covariances were included based on modification indices: IP28-IP44 (MI = 52.71, stability items) and IP62-IP60 (MI = 17.54, communication-recovery relationship). Configural invariance showed excellent fit (CFI = 0.997, TLI = 0.997, RMSEA = 0.033), confirming equivalent factor structure across groups. Factor correlations differed between groups (Comparison: 0.923; Autism: 0.808). Metric invariance maintained good fit (CFI = 0.992, RMSEA = 0.056) despite significant chi-square differences, χ^2^(16) = 41.16, *p* < .001. Scalar invariance showed good fit (CFI = 0.993, RMSEA = 0.047) with non-significant model differences, χ^2^(86) = 96.79, *p* = .200. Following Chen (2007), fit index changes supported invariance: ΔCFI values (−0.005, +0.0004) remained within acceptable thresholds (≤0.01). ΔRMSEA slightly exceeded recommendations for metric invariance (+0.023 > 0.015) but improved for scalar invariance (−0.008). The non-linear pattern (initial decline, then improvement) suggests that group differences exist in latent means rather than measurement properties, supporting equivalent construct measurement across populations.

### Step 3: Discriminative and Convergent Validity of Optimized Item Set Using Sample 1

**Figure 3.**
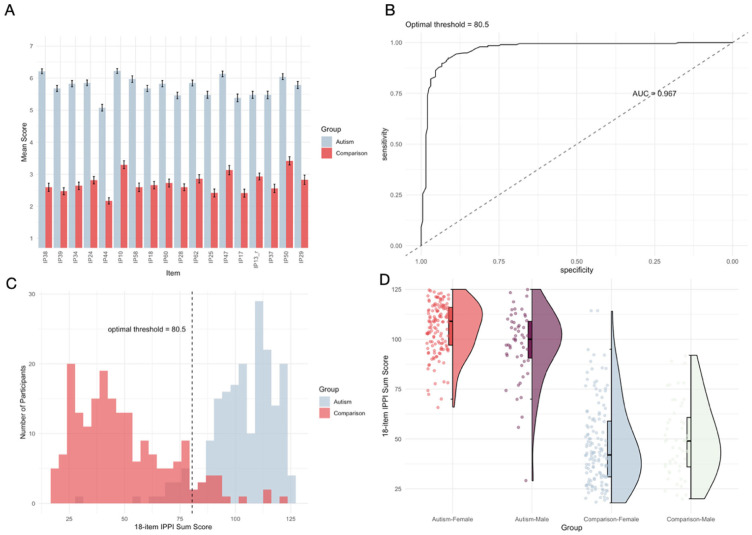
Optimized 18-item IPPI discrimination, classification performance, and group differences in Sample 1: (a) Mean scores (± SEM) of the Autism and the Comparison group for each selected IPPI item, sorted by discriminatory power. (b) ROC curve for the optimized IPPI version. (c) Distribution of 18-item IPPI version sum scores with classification threshold. (d) IPPI Sum Scores (boxplots, scatterplots, and density curve) of the Autism and Comparison group, separately for female and male participants.

The 18-item IPPI demonstrated exceptional discriminative ability between groups, with the Autism group scoring significantly higher (*M* = 103.43, *SD* = 17.34) than the Comparison group (*M* = 49.08, *SD* = 19.26), *t*(348.14) = 29.44, *p* < .001, Cohen’s *d* = 2.97 (Figure 3(a)). ROC analysis yielded excellent discriminative accuracy (AUC = 0.97; Figure 3(b)). The optimal threshold of 80.5 produced high sensitivity (0.92), specificity (0.91), and overall accuracy (0.92), correctly classifying 360 of 392 participants with only 32 misclassifications (15 false negatives and 17 false positives).

The IPPI showed strong convergent validity with established measures. Correlations with the AQ and BAP-Q were robust in both the Autism group (*r* = .67 and .78, respectively) and Comparison group (*r* = .79 and .82, respectively; all *p* < .001). These results indicate that the IPPI measures a construct closely related to but potentially distinct from those captured by existing instruments. The slightly stronger correlations in the Comparison group suggest the IPPI may be particularly sensitive to subclinical autistic traits. The AQ and BAP-Q were themselves highly correlated in both groups (Autism: *r* = .72, *p* < .001; Comparison: *r* = .85, *p* < .001), confirming the convergent validity of all three measures. Scatterplots are shown in Supplemental Figure S11.

To examine gender differences in IPPI scores, we fitted a linear regression model with IPPI sum scores as the dependent variable and diagnostic group, gender, and their interaction as predictors (IPPI score ~ group × gender). Due to violations of normality (Shapiro–Wilk *W* = 0.98, *p* < .001) and homoscedasticity (χ^2^ = 21.83, *p* < .001), we used heteroscedasticity-consistent standard errors (HC3) to ensure robust inference. The model explained 72.3% of the variance in IPPI scores, *R*² = .72, adjusted *R*² = .72, *F*(3, 374) = 324.61, *p* < .001. The regression revealed a significant interaction between gender and diagnostic group (*b* = 11.25, *SE* = 4.18, *t* = 2.69, *p* = .007, β = .14). Within the Autism group, females scored significantly higher (*M* = 105.56, *SD* = 12.60) than males (*M* = 97.08, *SD* = 18.10; *b* = −8.49, *SE* = 2.75, *t* = −3.08, *p* = .002, β = −.22, *d* = 0.54). In contrast, this pattern was reversed in the Comparison group, though the difference was not statistically significant, with males scoring slightly higher (*M* = 49.75, *SD* = 18.70) than females (*M* = 46.99, *SD* = 20.40, *d* = −0.14, *p* = .378; Figure 3(d)). The significant interaction effect indicates that gender influences the expression of autistic traits differently in autistic versus non-autistic individuals, with autistic females reporting higher levels of autistic traits than autistic males when measured with the IPPI.

### Step 4: Confirmatory Factor Analysis in Sample 2

To validate the factor structure identified through EFA, we conducted confirmatory factor analysis in the independent Sample 2 (*n* = 247 autistic participants). The two-factor model with “Prediction Integration and Interpretation” (12 items) and “Prediction Error Sensitivity and Stability Needs” (six items) demonstrated good fit (CFI = 0.987, TLI = 0.985, RMSEA = 0.050, SRMR = 0.064). All items loaded significantly on their respective factors, with standardized loadings ranging from 0.324 to 0.723 (Figure 2). The two error covariances included based on the EFA (between IP28 and IP44: 0.255, *p* < .001; between IP62 and IP60: −0.006, *p* = .885) showed a similar pattern to Sample 1, with the first remaining significant and the second non-significant. The correlation between factors was strong (*r* = .88), consistent with our previous findings.

The successful replication across samples with substantial demographic differences (Sample 2: older, more gender diverse) suggests the IPPI captures consistent constructs across varied autistic populations. McDonald’s omega reliability coefficients were 0.82 for “Prediction Integration and Interpretation” and 0.74 for “Prediction Error Sensitivity and Stability Needs,” indicating good internal consistency.

### Step 5: Extended Validation and Utility Assessment in Sample 3

**Figure 4.**
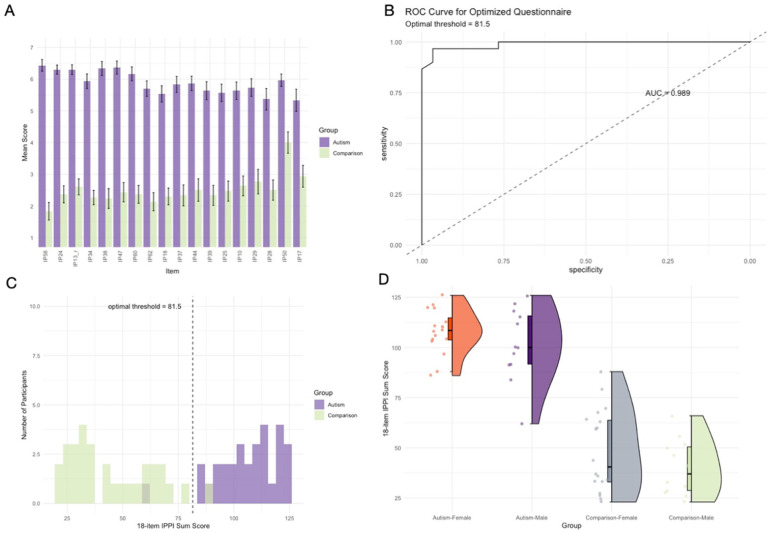
Extended Validation and Utility Assessment of 18-item IPPI version in Sample 3: (a) Mean scores (± SEM) of the Autism and the Comparison group for each selected IPPI item, sorted by discriminatory power. (b) ROC curve for the optimized IPPI version. (c) Distribution of sum scores with classification threshold. (d) IPPI Sum Scores (Boxplots, scatterplots, and density curve) of the Autism and Comparison group, separately for female and male participants.

We conducted further validation of the optimized IPPI in the in-lab Sample 3 (30 autistic, 30 non-autistic participants). The IPPI demonstrated strong discriminative ability: autistic participants scored significantly higher (*M* = 106.00, *SD* = 14.60) than non-autistic participants (*M* = 45.00, *SD* = 18.36), *t*(55.20) = 14.25, *p* < .001, Cohen’s *d* = 3.68 (Figure 4(a) and (c)). ROC analysis yielded excellent discriminative accuracy (AUC = 0.99) with optimal threshold 81.5 producing sensitivity 0.97, specificity 0.97, and accuracy 0.97 (Figure 4(b)). Only two of 60 participants were misclassified (one false negative and one false positive), replicating Sample 1 performance.

The IPPI showed robust convergent validity with established measures. Correlations with AQ and BAP-Q were significant in both groups (Autism: *r* = .61 and .75; Comparison: *r* = .76 and .83; all *p* < .001), replicating Sample 1 patterns. AQ-BAP correlations were also strong (Autism: *r* = .66; Comparison: *r* = .77).

Multiple regression analysis confirmed IPPI independence from cognitive abilities. The model explained 79% variance (*R*^2^ = .791, *p* < .001) with group membership as strongest predictor (β = −59.21, *p* < .001), while verbal IQ (β = −0.21, *p* = .111) and nonverbal IQ (β = −0.16, *p* = .419) were non-significant, indicating autism-specific rather than general cognitive differences.

The two-factor model demonstrated excellent fit in Sample 3 (CFI = 1.00, TLI = 1.00, RMSEA = 0.000, SRMR = 0.044). All items loaded significantly and strongly on their respective factors, with standardized loadings ranging from 0.658 to 0.957. The correlation between factors was very strong (*r* = .98). These results provide additional confirmation of the factor structure’s validity, though the small sample size (*n* = 60) may contribute to the perfect fit indices and should be interpreted cautiously.

To examine potential gender differences in IPPI scores, we fitted a linear regression model with IPPI total scores as the dependent variable and group, gender, and their interaction as predictors (IPPI score ~ group × gender). The model accounted for 78.2% of the variance in IPPI scores, *R*^2^ = .782, adjusted *R*^2^ = .770, *F*(3, 54) = 64.48, *p* < .001. The analysis revealed no significant interaction between gender and group (*b* = −1.90, *SE* = 8.85, *t* = −0.22, *p* = .831), indicating that gender effects were consistent across diagnostic groups. The main effect of group was highly significant (*b* = −59.60, *SE* = 5.70, *t* = −10.46, *p* < .001), confirming substantial differences between Autism and Comparison groups. However, no significant main effect of gender emerged (*b* = −6.29, *SE* = 6.33, *t* = −0.99, *p* = .325). The absence of a significant interaction suggests that the IPPI measures predictive processing characteristics consistently across genders in both autistic and non-autistic populations (Figure 4(d)).

## Discussion

This study developed and psychometrically evaluated the IPPI, a comprehensive self-report measure assessing predictive processing characteristics and their daily consequences in autistic and non-autistic adults. Developed through community-led participatory research, the IPPI demonstrated exceptional psychometric properties, achieving excellent discriminative validity (AUC > 0.97) and strong convergent validity with established measures. Several methodological features enhance confidence in these findings. The participatory development approach represents a methodological advance ensuring authentic autistic experiences, the multi-sample validation across online and laboratory settings demonstrates robustness across contexts, and the network-based optimization provides a data-driven approach while maintaining theoretical coherence.

Network-based item selection successfully reduced the initial 65-item version to an efficient 18-item scale while maintaining excellent reliability (α = .97) with improved discriminative power. Exploratory, data-driven factor analysis revealed a stable two-factor structure: “Prediction Integration and Interpretation” (12 items), capturing challenges in integrating sensory and social inputs with predictive models, and “Prediction Error Sensitivity and Stability Needs” (six items), reflecting the need for environmental predictability and distress responses to unexpected changes.

Importantly, the IPPI items capture outcomes that are theoretically grounded in predictive processing mechanisms while remaining accessible to subjective report. For instance, Item IP58 (“Often I feel overwhelmed by sounds, smells or colours”) does not directly measure precision weighting, which would be inaccessible to introspection, but rather captures the subjective experience that predictive processing theory proposes would result from excessive precision assigned to bottom-up sensory inputs (Pellicano & Burr, 2012). Similarly, Item IP18 (“Other people misinterpret my facial expressions or gestures”) reflects the observable consequence of divergent generative models between communication partners (Friston & Frith, 2015). By focusing on subjective manifestations of predictive processing differences rather than prediction accuracy per se, the IPPI bridges theoretical frameworks with lived experience (Supplemental Table S10 provides the complete theoretical rationale for how each item relates to predictive processing constructs).

The IPPI, assessing theoretically grounded experiential outcomes, represents a significant theoretical advance for understanding the psychological challenges that autistic people face (Stark et al., 2021). This measurement approach distinguishes it from measures assessing prediction accuracy directly (PRE-Q; O’Brien et al., 2025) and earlier trait measures developed without an integrated theoretical framework (e.g., AQ; Baron-Cohen et al., 2001).

A note of caution regarding the interpretation of IPPI scores is warranted. As the measure’s name reflects, the IPPI was designed to capture introspective reports of experiences that predictive processing theory proposes as characteristic. The “Introspective” qualifier is deliberate: items assess subjective manifestations theoretically linked to predictive processing differences, not predictive processing mechanisms themselves. Accordingly, a high IPPI score should not be interpreted as confirming that an individual has predictive processing differences at a mechanistic level. Like other theory-driven measures, the IPPI operationalizes a theoretical framework in terms accessible to subjective report, and the theoretical links between items and predictive processing mechanisms remain hypothetical rather than directly validated. Establishing such direct links will require future research examining relationships between IPPI scores and objective indicators of predictive processing, such as neural prediction error responses or behavioral tasks. Researchers are encouraged to interpret the IPPI as a measure of experiences that are consistent with predictive processing accounts of autism, rather than as a direct index of predictive processing itself.

The IPPI’s two-factor structure covers content from multiple competing predictive processing accounts. Factor 1 items capture experiences consistent with hypo-priors accounts, emphasizing reduced reliance on cognitive predictions and difficulties integrating prior expectations with incoming sensory and social input (Bolis et al., 2017; Pellicano & Burr, 2012) and precision weighting theories (Van de Cruys et al., 2014), while Factor 2 items reflect temporal prediction difficulties (Sinha et al., 2014) and overly precise sensitivity to prediction errors (Van de Cruys et al., 2014). Rather than favoring a single mechanism, the IPPI provides a measurement framework for testing which theoretical accounts best explain individual differences in predictive processing experiences.

Several items further effectively operationalize theoretical concepts from interpersonal predictive processing (Friston & Frith, 2015), such as prediction errors arising from divergent generative models between communication partners (e.g., “Others don’t understand me,” “People misinterpret my expressions”). In addition, the content of these items reflects key assumptions of the concept of the double empathy problem (Milton, 2012), which proposes that communication difficulties between autistic and non-autistic individuals may arise from mutual misunderstanding due to different cognitive styles, rather than from unidirectional deficits in autistic individuals. Empirical evidence indicates that non-autistic individuals face comparable difficulties when inferring mental states of autistic individuals (Sheppard et al., 2016). Accordingly, the IPPI items addressing misunderstandings and misinterpretations account for this bidirectional nature of predictive processing challenges in social interaction. The two-factor structure was confirmed across independent samples and demonstrated measurement invariance between groups and remained independent of general intelligence. While the full 65-item version demonstrated strong internal consistency (α = .99) and discriminative validity (AUC = 0.96; Supplemental Material S8), it has not undergone factor structure validation or measurement invariance testing. The 18-item optimized IPPI represents the validated instrument for research and clinical use. However, the comprehensive 65-item pool may warrant future investigation for potential applications requiring item redundancy or for exploring differential item functioning across neurotypes, though such applications would require appropriate psychometric validation.

Several limitations should be acknowledged. The convenience sampling strategy may have introduced selection bias, including reversed sex ratios, higher education levels, and later age of diagnosis compared to population-based samples (Rødgaard et al., 2022). While these characteristics may enhance generalizability compared to clinic-based samples and align with recent calls for more inclusive autism research approaches, they limit representativeness of the broader autism population. However, the combination of online and laboratory-based samples with controlled matching for cognitive abilities helps address some of these sampling concerns. Future research should examine the IPPI’s temporal stability through test–retest reliability studies and explore its sensitivity to change in longitudinal designs. Investigation of relationships between IPPI scores and objective measures of predictive processing (e.g., neuroimaging, behavioral tasks) could further establish construct validity. In addition, the PRE-Q (O’Brien et al., 2025) was not available during our data collection period. Future research should examine convergent validity between the IPPI and PRE-Q, as these measures capture complementary aspects of predictive processing experiences. Finally, while we observed gender differences in IPPI scores within the autistic group, we did not conduct measurement invariance testing by gender due to sample size constraints. Future research should examine whether the IPPI demonstrates equivalent measurement properties across genders before drawing firm conclusions about gender-specific patterns in predictive processing experiences.

The measure’s potential utility in intervention research, such as monitoring changes following anxiety treatments or environmental modifications, warrants exploration. Future research could investigate whether IPPI profiles inform personalized intervention strategies, such as whether individuals with high scores on Factor 2 (Prediction Error Sensitivity) benefit more from predictability-enhancing support. The IPPI revealed different gender patterns compared with traditional measures like the AQ, with autistic females scoring higher than autistic males in Sample 1, though this pattern was not replicated in Sample 3. These preliminary findings suggest the IPPI may capture aspects of autism that are either more pronounced in females or less influenced by male-biased conceptualizations of autism. However, without measurement invariance testing across genders, these patterns should be interpreted cautiously and warrant further investigation to understand whether they reflect distinct predictive processing experiences across genders or measurement artifacts.

In sum, the IPPI provides a validated tool for assessing internal predictive processing experiences, advancing theory-driven autism research that centers autistic perspectives.

## Supplemental Material

sj-pdf-1-aut-10.1177_13623613261443728 – Supplemental material for Participatory Development and Psychometric Evaluation of the Introspective Predictive Processing Inventory: A Self-Report Measure for Autistic and Non-Autistic Adults Supplemental material, sj-pdf-1-aut-10.1177_13623613261443728 for Participatory Development and Psychometric Evaluation of the Introspective Predictive Processing Inventory: A Self-Report Measure for Autistic and Non-Autistic Adults by Marik Roos, Hannah Storm, Lucie Zimmer and Tobias Schuwerk in Autism

## Appendix

**Appendix 1.** 18-item Introspective Predictive Processing Inventory (IPPI; English version). *Optimized short version of the IPPI. Respondents rate each statement on a 7-point Likert-type scale, with only the endpoints labeled: “do not agree at all / very atypical for me” (1) and “agree very strongly/very typical for me” (7). The total score is calculated as the sum of all item responses (possible range: 18–126). Item 13 is reverse-coded*. An interactive version of the validated 18-item IPPI is freely accessible at https://ippi-tool.netlify.app/. The tool runs entirely in the user’s browser and does not collect or store any data.

Item	Statement
IP10	I often have the feeling that others don’t understand me.
IP13_r	People’s facial expressions are very reliable sources of information.
IP17	I like to wear the same outfit over and over again to make me feel at ease.
IP18	Other people misinterpret my facial expressions or gestures.
IP24	I have a hard time guessing how another person might understand the things I say.
IP25	It is calming to repeat movements over and over again.
IP28	It ruins my whole day, if things don’t happen as expected.
IP29	I don’t like talking on the phone because I can’t tell when the other person is going to say something.
IP34	When people ask me questions, I often don’t know what they are actually getting at and how exactly I should answer.
IP37	It would stress me out if I had to spontaneously take a different route to school/work.
IP38	It takes a lot of strength for me to appear “normal” to others.
IP39	I often find it difficult to determine which information is relevant to me at the moment.
IP44	I am completely upset if just one little thing doesn’t go according to plan.
IP47	If several people are talking at the same time, I can’t really listen to any of them.
IP50	During a stressful situation, I often don’t realize how stressed I actually am and why exactly.
IP58	Often I feel overwhelmed by sounds, smells, or colors.
IP60	It takes a long time for me to recover from everyday challenges.
IP62	For me, communication is often associated with frustration and/or stress.

## References

[bibr1-13623613261443728] Angeletos Chrysaitis N. Seriès P . (2023). 10 years of Bayesian theories of autism: A comprehensive review. Neuroscience & Biobehavioral Reviews, 145, Article 105022. 10.1016/j.neubiorev.2022.105022 36581168

[bibr2-13623613261443728] Baron-Cohen S. Wheelwright S. Skinner R. Martin J. Clubley E. (2001). The autism-spectrum quotient (AQ): Evidence from Asperger syndrome/high-functioning autism, males and females, scientists and mathematicians. Journal of Autism and Developmental Disorders, 31, 5–17. 10.1023/A:1005653411471 11439754

[bibr3-13623613261443728] Bolis D. Balsters J. Wenderoth N. Becchio C. Schilbach L. (2017). Beyond autism: Introducing the dialectical misattunement hypothesis and a Bayesian account of intersubjectivity. Psychopathology, 50(6), 355–372. 10.1159/000484353 29232684

[bibr4-13623613261443728] Cannon J. O’Brien A. M. Bungert L. Sinha P. (2021). Prediction in autism spectrum disorder: A systematic review of empirical evidence. Autism Research, 14(4), 604–630. 10.1002/aur.2482 33570249 PMC8043993

[bibr5-13623613261443728] Chen F. F. (2007). Sensitivity of goodness of fit indexes to lack of measurement invariance. Structural Equation Modeling: A Multidisciplinary Journal, 14(3), 464–504. 10.1080/10705510701301834

[bibr6-13623613261443728] Clark A. (2013). Whatever next? Predictive brains, situated agents, and the future of cognitive science. Behavioral and Brain Sciences, 36(3), 181–204. 10.1017/S0140525X12000477 23663408

[bibr7-13623613261443728] Costello A. B. Osborne J. (2005). Best practices in exploratory factor analysis: Four recommendations for getting the most from your analysis. Practical Assessment, Research, and Evaluation, 10(1), Article 7. 10.7275/jyj1-4868

[bibr8-13623613261443728] den Houting J. Higgins J. Isaacs K. Mahony J. Pellicano E . (2021). ‘I’m not just a guinea pig’: Academic and community perceptions of participatory autism research. Autism, 25(1), 148–163. 10.1177/1362361320951696 32854511

[bibr9-13623613261443728] Freitag C. M. Retz-Junginger P. Retz W. Seitz C. Palmason H. Meyer J. Rösler M. von Gontard A. (2007). Evaluation der deutschen Version des Autismus-Spektrum-Quotienten (AQ)—die Kurzversion AQ-k [German adaptation of the Autism-Spectrum Quotient (AQ): Evaluation and short version AQ-k]. Zeitschrift für Klinische [Psychologie und Psychotherapie], 36(4), 280–289. 10.1026/1616-3443.36.4.280

[bibr10-13623613261443728] Friston K. Frith C. (2015). A duet for one. Consciousness and Cognition, 36, 390–405. 10.1016/j.concog.2014.12.003 25563935 PMC4553904

[bibr11-13623613261443728] Holroyd C. B. Coles M. G. H. (2002). The neural basis of human error processing: Reinforcement learning, dopamine, and the error-related negativity. Psychological Review, 109(4), 679–709. 10.1037/0033-295X.109.4.679 12374324

[bibr12-13623613261443728] Hull L. Mandy W. Lai M. C. Baron-Cohen S. Allison C. Smith P. Petrides K. V. (2019). Development and validation of the Camouflaging Autistic Traits Questionnaire (CAT-Q). Journal of Autism and Developmental Disorders, 49, 819–833. 10.1007/s10803-018-3792-6 30361940 PMC6394586

[bibr13-13623613261443728] Hurley R. S. E. Losh M. Parlier M. Reznick J. S. Piven J. (2007). The broad autism phenotype questionnaire. Journal of Autism and Developmental Disorders, 37(9), 1679–1690. 10.1007/s10803-006-0299-3 17146701

[bibr14-13623613261443728] Jorgensen T. D. Pornprasertmanit S. Schoemann A. M. Rosseel Y. (2021). semTools: Useful tools for structural equation modeling (R package version 05-6). https://CRAN.R-project.org/package=semTools

[bibr15-13623613261443728] Koster-Hale J. Saxe R. (2013). Theory of mind: A neural prediction problem. Neuron, 79(5), 836–848. 10.1016/j.neuron.2013.08.020 24012000 PMC4041537

[bibr16-13623613261443728] Lehrl S. (2005). Mehrfachwahl-Wortschatz-Intelligenztest MWT-B. Spitta-Verlag.

[bibr17-13623613261443728] Leiner D. J. (2019). SoSci survey (Version 3.2.30) [Computer software]. https://www.soscisurvey.de

[bibr18-13623613261443728] Milton D. E. M. (2012). On the ontological status of autism: The ‘double empathy problem’. Disability & Society, 27(6), 883–887. 10.1080/09687599.2012.710008

[bibr19-13623613261443728] O’Brien A. M. May T. A. Koskey K. L. K. Bungert L. Cardinaux A. Cannon J. Treves I. N. D’Mello A. M. Joseph R. M. Li C. Diamond S. Gabrieli J. D. E. Sinha P. (2025). Development of a self-report measure of prediction in daily life: The Prediction-Related Experiences Questionnaire. Journal of Autism and Developmental Disorders, 55, 2550–2565. 10.1007/s10803-024-06379-2 38713266 PMC12167306

[bibr20-13623613261443728] Pellicano E. Burr D. (2012). When the world becomes ‘too real’: A Bayesian explanation of autistic perception. Trends in Cognitive Sciences, 16(10), 504–510. 10.1016/j.tics.2012.08.009 22959875

[bibr21-13623613261443728] Pons P. Latapy M. (2005). Computing communities in large networks using random walks. In Yolum p. Güngör T. Gürgen F. Özturan C. (Eds.), Computer and Information Sciences—ISCIS 2005 (Lecture Notes in Computer Science, Vol. 3733, pp. 284–293). Springer. 10.1007/11569596_31

[bibr22-13623613261443728] Posit Team. (2024). RStudio: Integrated development environment for R (Version 2024.12.0+467). Posit Software, PBC. http://www.posit.co/

[bibr23-13623613261443728] Rødgaard E. M. Jensen K. Miskowiak K. W. Mottron L. (2022). Representativeness of autistic samples in studies recruiting through social media. Autism Research, 15(8), 1447–1456. 10.1002/aur.2777 35809003 PMC9541916

[bibr24-13623613261443728] R Core Team. (2024). R (Version 4.4.2): A language and environment for statistical computing. R Foundation for Statistical Computing. https://www.R-project.org

[bibr25-13623613261443728] Revelle W. (2024). psych: Procedures for psychological, psychometric, and personality research (R package version 2412). https://CRAN.R-project.org/package=psych

[bibr26-13623613261443728] Robin X. Turck N. Hainard A. Tiberti N. Lisacek F. Sanchez J.-C. Müller M. (2011). pROC: An open-source package for R and S+ to analyze and compare ROC curves. BMC Bioinformatics, 12, Article 77. 10.1186/1471-2105-12-77 PMC3068975 21414208

[bibr27-13623613261443728] Roos M. (2020, March 19–20). Das Introspektive Predictive Processing Inventar (IPPI): Erste Evaluation von Validität und Reliabilität eines neuen Messinstruments [The Introspective Predictive Processing Inventory (IPPI): First evaluation of validity and reliability of a new measurement instrument] [Conference poster]. Wissenschaftliche Tagung Autismus Spektrum, Göttingen, Germany.

[bibr28-13623613261443728] Rosseel Y. (2012). lavaan: An R package for structural equation modeling. Journal of Statistical Software, 48, 1–36. 10.18637/jss.v048.i02

[bibr29-13623613261443728] Schuwerk T. Kaltefleiter L. J. Au J. Q. Hoesl A. Stachl C. (2019). Enter the wild: Autistic traits and their relationship to mentalizing and social interaction in everyday life. Journal of Autism and Developmental Disorders, 49, 4193–4208. 10.1007/s10803-019-04134-6 31273579

[bibr30-13623613261443728] Schwartz S. Susser E. (2011). The use of well controls: An unhealthy practice in psychiatric research. Psychological Medicine, 41(6), 1127–1131. 10.1017/S0033291710001595 20810003

[bibr31-13623613261443728] Sheppard E. Pillai D. Wong G. T. Ropar D. Mitchell P. (2016). How easy is it to read the minds of people with autism spectrum disorder? Journal of Autism and Developmental Disorders, 46(4), 1247–1254. 10.1007/s10803-015-2662-8 26603886

[bibr32-13623613261443728] Sinha P. Kjelgaard M. M. Gandhi T. K. Tsourides K. Cardinaux A. L. Pantazis D. Diamond S. P. Held R. M. (2014). Autism as a disorder of prediction. Proceedings of the National Academy of Sciences of the United States of America, 111(42), 15220–15225. 10.1073/pnas.1416797111 PMC4210351 25288765

[bibr33-13623613261443728] Stark E. Stacey J. Mandy W. Kringelbach M. L. Happé F. (2021). Autistic cognition: Charting routes to anxiety. Trends in Cognitive Sciences, 25(7), 571–581. 10.1016/j.tics.2021.03.014 33958281

[bibr34-13623613261443728] Van de Cruys S. Evers K. Van der Hallen R. Van Eylen L. Boets B. de-Wit L. Wagemans J. (2014). Precise minds in uncertain worlds: Predictive coding in autism. Psychological Review, 121(4), 649–675. 10.1037/a0037665 25347312

[bibr35-13623613261443728] Weiß R. H. (2006). Grundintelligenztest Skala 2—Revision (CFT 20-R) [Culture Fair Intelligence Test Scale 2—Revision (CFT 20-R)]. Hogrefe.

[bibr36-13623613261443728] Zimmer L. Richardson H. Pletti C. Paulus M. Schuwerk T. (2025). Predictive responses in the theory of mind network: A comparison of autistic and non-autistic adults. Cortex, 187, 159–171. 10.1016/j.cortex.2025.04.006 40373360

