# Supplementary material for: Participatory Development and Psychometric Evaluation of the Introspective Predictive Processing Inventory: A Self-Report Measure for Autistic and Non-Autistic Adults
Source: Autism. 2026 May 3;30(6):1593–607. doi: 10.1177/13623613261443728 (PMC13187222; doi:10.1177/13623613261443728)
Supplement: sj-pdf-1-aut-10.1177_13623613261443728 – Supplemental material for Participatory Development and Psychometric Evaluation of the Introspective Predictive Processing Inventory: A Self-Report Measure for Autistic and Non-Autistic Adults [file sj-pdf-1-aut-10.1177_13623613261443728.pdf]

**Supplemental Material**

This document contains supplemental material of the research report:

Roos, M., Strom, H., Zimmer, L., & Schuwerk, T. (2026). Development and Psychometric Evaluation of the Introspective Predictive Processing Inventory (IPPI): A Self-Report Measure for Autistic and Non-autistic Adults. *Autism.*

## S1. Countries of Residence

Overview of the participants' countries of residence (pooled sample of N = 443 autistic and N = 287 non-autistic adults).

| Current Residency | Autism Group | Comparison Group |
|---:|---:|---:|
| Australia | 2 | 0 |
| Austria | 12 | 10 |
| Belgium | 1 | 0 |
| Canada | 4 | 0 |
| Croatia | 2 | 0 |
| Denmark | 2 | 0 |
| Germany | 316 | 297 |
| Greece | 0 | 1 |
| Ireland | 4 | 0 |
| Israel | 1 | 0 |
| Italy | 1 | 0 |
| Liechtenstein | 0 | 1 |
| Netherlands | 5 | 0 |
| New Zealand | 2 | 0 |
| No Answer | 4 | 3 |
| Peru | 1 | 0 |
| Poland | 0 | 1 |
| Serbia | 1 | 0 |
| Spain | 2 | 3 |
| Sweden | 1 | 1 |
| Switzerland | 2 | 0 |
| United Kingdom | 97 | 0 |
| United States | 13 | 0 |

**S2. Diagnosis Verification and Check for Sample Differences**

Autistic participants who agreed to the optional verification of their autism diagnosis were offered two options at the end of the study. First, they could provide their email address—stored separately from the study data—to schedule an approximately five-minute online meeting via the video therapy platform REDconnect (certified by German health insurance providers for teletherapy). In this unrecorded meeting, participants were asked to briefly hold a document confirming their diagnosis up to the webcam, and a study team member documented the presence of the diagnosis without collecting any personal data. Alternatively, participants could upload an image or PDF of the diagnostic document to a secure LMU Munich server via a provided link. Access to this server was restricted to project personnel, and once the existence of the diagnosis was noted, the uploaded file was immediately and permanently deleted.

In preliminary analyses conducted on the online-recruited samples (Samples 1 and 2, n = 443), we examined potential differences between participants with a formal diagnosis (n = 189, 42.66 % of the pooled sample), those with a verified formal diagnosis (n = 165, 37.25 % of the pooled sample), and self-identifying autistic participants (n = 89, 20.09 % of the pooled sample). First, we assessed whether participants with a formal diagnosis (verified by the study team or not) differed from self-identifying autistic participants in their scores on the IPPI sum score (initial 65-item version), our key variable of interest. No significant difference was found, $W = 14869$, $p = .413$, $r = .04$. Second, we examined whether participants with a formal diagnosis differed from those with a verified formal diagnosis in their IPPI sum scores. Again, no significant difference was observed, $W = 14874$, $p = .454$, $r = .04$. Because these groups did not differ in their IPPI sum scores, we collapsed them for the subsequent analyses.

We further examined whether participants differed in their IPPI sum scores depending on whether they completed the English or German version. In the Autism group, IPPI sum scores did not differ significantly between the English and German versions, $W = 22{,}127$, $p = .770$, $r = .01$. Similarly, in the Comparison group, no significant difference was found, $W = 2{,}469$, $p = .171$, $r = .08$. Consequently, we did not differentiate between the two language versions in the subsequent analyses.

**Table S3.**

*Prevalence of further mental health diagnoses by sample. Percentages listed for 0,1,2, or 3+ comorbid diagnoses.*

| | Autism Group | Comparison Group |
|---|---|---|
| ***Sample 1*** | | |
| n = 0 | 18.37 % | 71.94 % |
| n = 1 | 22.45 % | 18.37 % |
| n = 2 | 26.02 % | 7.65 % |
| n ≥ 3 | 33.16 % | 2.04 % |
| ***Sample 2*** | | |
| n = 0 | 20.65 % | - |
| n = 1 | 21.05 % | - |
| n = 2 | 25.91 % | - |
| n ≥ 3 | 32.39 % | - |
| ***Sample 3*** | | |
| n = 0 | 26.70 % | -[1] |
| n = 1 | 16.70 % | - |
| n = 2 | 23.30 % | - |
| n ≥ 3 | 33.30 % | - |

*Note.* [1] no data on mental health diagnoses were collected for non-autistic participants in Sample 3

## S4. Matching Procedure Sample 1

The convenience sampling in the present study resulted in an Autism and a Comparison group that were unbalanced in size, age and gender. Thus, from the overall pool of online-recruited participants, we drew the largest possible subsample of autistic and non-autistic individuals such that the ASC and comparison groups were matched for age and gender, using nearest-neighbor propensity score matching (PSM). The matching was implemented using the matchit function from the MatchIt R package (Ho et al., 2011), applying nearest-neighbor matching with a 1:1 ratio. Matching was conducted on age with a caliper of 0.3 standard

deviations and exact matching on gender (Austin, 2011; Rosenbaum & Rubin, 1983). This approach ensured that each participant from the Autism group was paired with the most similar participant from the Comparison group within the same gender and age range, thereby reducing selection bias and improving group comparability. This procedure resulted in a sample of 196 participants in the Autism and 196 participants in the Comparison group.

## S5. Initial 65-item Version of the IPPI in German

| Item | Statement |
|---|---|
| IP01 | Ich brauche sehr lange, um mich an Veränderungen zu gewöhnen. |
| IP02 | Wenn mich jemand bitten würde, etwas noch einmal mit anderen Worten zu wiederholen, würde ich mich unwohl fühlen, weil sich dadurch vielleicht die Bedeutung des Inhalts ändert. |
| IP03 | Ich habe oft das Gefühl, dass mir Dinge nicht richtig erklärt werden. |
| IP04 | Ich fühle mich oft überfordert von der Anzahl an Möglichkeiten, wie eine Situation ausgehen wird. |
| IP05 | Ich spüre nicht so recht, wo mein Körper aufhört, wenn ich nicht bewusst von etwas anderem berührt werde (z. B. einer Bettdecke). |
| IP06_r | Wenn meine Strategie zur Lösung eines Problems nicht hilft, denke ich mir einfach schnell eine neue aus. |
| IP07 | Wenn sich die Fahrpläne öffentlicher Verkehrsmittel in meiner Nachbarschaft ändern, bringt mich das völlig aus dem Konzept. |
| IP08 | Vor einer unbekannten Situation spiele ich im Kopf alle möglichen Szenarien durch, auch wenn diese sehr unwahrscheinlich sind. |
| IP09 | Soziale Situationen sind meistens sehr komplex und unvorhersehbar. |
| IP10 | Ich habe oft das Gefühl, dass andere mich nicht verstehen. |
| IP11 | In der Schule habe ich eher viele einzelne Details verinnerlicht als wirklich die zugrunde liegenden Zusammenhänge zu verstehen. |
| IP12 | Ich fühle mich verloren und unsicher, wenn Situationen anders ausgehen als erwartet. |
| IP13_r | Die Gesichtsausdrücke von Menschen sind sehr zuverlässige Informationsquellen. |
| IP14 | Jeden Tag dasselbe zu tun würde mir ein Gefühl von Sicherheit geben. |
| IP15 | Ich werde oft von plötzlichen Geräuschen oder Bewegungen aus meiner Konzentration gerissen, die andere Personen auszublenden scheinen. |
| IP16 | Ich fühle mich oft orientierungslos und überfordert. |
| IP17 | Ich trage sehr oft dasselbe Outfit, um mich sicher zu fühlen. |
| IP18 | Andere Menschen interpretieren meine Gesichtsausdrücke oder Gesten falsch. |
| IP19 | Wenn ich bei einem Spiel oder der Bearbeitung einer Aufgabe merke, dass meine gewohnte Strategie nicht aufgeht, fühle ich mich frustriert und orientierungslos. |
| IP20 | Ich habe oft die Erfahrung gemacht, dass andere mich zurückweisen, wenn ich meinen Bedürfnissen und Gefühlen entsprechend handle. |
| IP21_r | Wenn ein unerwartetes Ereignis eintritt, improvisiere ich einfach. |
| IP22 | Wenn ich eine Auswahl aus mir unbekannten Dingen treffen muss (z. B. Speisen in einem Restaurant), fühle ich mich schnell überfordert. |
| IP23 | Manchmal plappere ich vor mich hin, um mich der Dinge zu vergewissern, die ich gerade tue. |

IP24 Ich kann nur schlecht einschätzen, wie mein Gegenüber die Dinge versteht, die ich sage.

IP25 Bewegungen immer wieder zu wiederholen beruhigt mich.

IP26 Ich habe das Gefühl, ich kann den Erwartungen anderer nicht gerecht werden.

IP27 Wenn jemand mit mir flirten würde, würde ich es nicht bemerken.

IP28 Wenn Dinge nicht wie erwartet eintreten, ist mein ganzer Tag ruiniert.

IP29 Ich telefoniere nicht gerne, weil ich nicht weiß, wann die andere Person was sagen wird.

IP30 Nach einer stressigen Situation brauche ich oft mehrere Stunden, um mich zu regenerieren.

IP31 Wenn man mich nach konkreten Beispielen für abstrakte Konstrukte fragt, fallen mir oft keine ein (z. B. wie beim Beantworten dieser Frage).

IP32 Ich verbringe manchmal mehrere Stunden täglich damit, alle möglichen Ausgänge von Situationen im Kopf durchzuspielen, auch wenn diese schon in der Vergangenheit liegen.

IP33 Ich war als Kind oft frustriert und/oder deprimiert.

IP34 Wenn Menschen mir Fragen stellen, weiß ich oft nicht, worauf sie eigentlich hinauswollen und wie ich genau antworten muss.

IP35 Ich kann mich beim Auspacken eines Geburtstagsgeschenks viel mehr freuen, wenn ich schon weiß, was drin ist.

IP36 Wenn ich nicht genau weiß, was als nächstes passieren wird, werde ich nervös.

IP37 Es würde mich stressen, wenn ich spontan einen anderen Weg zur Schule/Arbeit nehmen müsste.

IP38 Es kostet mich sehr viel Kraft, auf andere „normal“ zu wirken.

IP39 Es fällt mir oft schwer zu entscheiden, welche Informationen gerade relevant für mich sind.

IP40 Ich kann Emotionen (z. B. Freude oder Trauer) viel stärker fühlen, wenn ich zu Hause bin.

IP41 Ich habe als Kind oft nicht verstanden, warum Erwachsene mit mir schimpfen.

IP42 Im Alltag funktionieren zu müssen kostet mich viel Energie.

IP43 Wenn ich über ein für mich interessantes Thema spreche, merke ich oft, dass ich immer dieselben Formulierungen verwende.

IP44 Es bringt mich komplett aus der Fassung, wenn nur eine Kleinigkeit nicht nach Plan läuft.

IP45 Ich finde es sehr viel leichter, meine Gefühle wahrzunehmen, wenn ich in einer gewohnten Umgebung bin.

IP46 Ich bemerke oft Dinge (z. B. Geräusche), die andere Leute nicht wahrnehmen.

IP47 Wenn mehrere Personen gleichzeitig reden, kann ich keiner davon zuhören.

IP48 Ich habe das Gefühl, dass ich mich in der Welt da draußen auf nichts verlassen kann.

IP49 Ich habe ein großes Bedürfnis nach Halt und Sicherheit.

IP50 Während einer Stresssituation merke ich oft gar nicht, wie gestresst ich eigentlich bin und warum genau.

IP51 Eine romantische Beziehung einzugehen ist/wäre unsicher und/oder stressig für mich.

IP52 Wenn ich den Text für ein Theaterstück lernen müsste, würde ich mir eher die einzelnen Sätze und Stichwörter merken als die Handlung als Ganzes zu verinnerlichen.

IP53 Wenn ich eine Theorie zu einem Sachverhalt habe, bei der sich eine Variable als falsch herausstellt, stelle ich lieber eine neue Theorie auf als die alte zu aktualisieren.

IP54 Ich erledige Aufgaben gern auf dieselbe Art und Weise, um mich sicher zu fühlen.

IP55_r Wenn ich mit anderen in einem Raum bin, kann ich mühelos der Konversation folgen, die mich gerade am meisten interessiert.

IP56 Es kann Jahre dauern, bis ich einem Menschen so sehr vertraue, dass ich mich in seiner Gesellschaft ungezwungen fühle.

IP57 Ich habe oft negative Gefühle, wenn ich nicht weiß, was von mir erwartet wird.

IP58 Oft fühle ich mich von Geräuschen, Gerüchen oder Farben regelrecht überflutet.

IP59 Wenn jemand mir eine Geschichte erzählt, weiß ich meistens nicht, wo sie hinführen wird.

| | |
|---|---|
| IP60 | Ich brauche sehr lange, um mich von alltäglichen Herausforderungen zu erholen. |
| IP61 | Wenn ich etwas erklärt bekomme, brauche ich viele Beispiele, um das Gefühl zu haben, es wirklich zu verstehen. |
| IP62 | Kommunikation ist für mich oft mit Frust und/oder Stress verbunden. |
| IP63 | Manchmal habe ich das Gefühl, in einer Situation einfach alle Informationen aufzunehmen und erst hinterher bewerten zu können, welche wichtig und unwichtig für mich waren. |
| IP64 | Es reizt mich nicht, meine Lieblingsband live zu hören, weil ich ihre Musik am liebsten so mag, wie sie auf der CD klingt. |
| IP65 | Auf den ersten Bissen einer Mahlzeit reagiere ich oft sehr sensibel, so als müsste sich mein Mund erst daran gewöhnen. |

**S6. Initial 65-item Version of the IPPI in English**

| Item | Statement |
|---|---|
| IP01 | It takes me a long time to adapt to changes. |
| IP02 | If someone asked me to repeat something again in different words, I would feel uncomfortable because it might change the meaning of the content. |
| IP03 | I often feel that things are not properly explained to me. |
| IP04 | I often feel overwhelmed by the number of ways a situation might turn out. |
| IP05 | I don't really feel my body unless I consciously come into contact with something else (like, for example, a blanket). |
| IP06_r | If my strategy for solving a problem doesn't work out, I just quickly think up a new one. |
| IP07 | When public transport timetables change in my neighbourhood, it completely puts me off my stride. |
| IP08 | If I am facing an unknown situation, I run through all possible outcomes in my head, even if they are very unlikely. |
| IP09 | Social situations are usually very complex and unpredictable. |
| IP10 | I often have the feeling that others don't understand me. |
| IP11 | At school, I tended to internalise specific details rather than really understand the underlying connections. |
| IP12 | I feel lost and insecure when situations turn out differently than expected. |
| IP13_r | People's facial expressions are very reliable sources of information. |
| IP14 | Doing the same thing every day would give me a sense of security. |
| IP15 | My concentration is often disturbed by sudden noises or movements that other people seem to block out. |
| IP16 | I often feel disoriented and overwhelmed. |
| IP17 | I like to wear the same outfit over and over again to make me feel at ease. |
| IP18 | Other people misinterpret my facial expressions or gestures. |
| IP19 | If my usual strategy is not working out when playing a game or performing a task, I feel frustrated and disoriented. |
| IP20 | I have often experienced that others reject me if I act according to my needs and feelings. |

IP21_r When I find myself in an unexpected situation, I simply switch to improvising.

IP22 When I have to decide what to choose among things I don't know (e.g. food in a restaurant), I feel easily overwhelmed.

IP23 I sometimes babble away in a commenting mode to reassure myself of the things I am doing at the moment.

IP24 I have a hard time guessing how another person might understand the things I say.

IP25 It is calming to repeat movements over and over again.

IP26 I feel I can't live up to the expectations of others.

IP27 If someone flirted with me, I wouldn't notice.

IP28 It ruins my whole day, if things don't happen as expected.

IP29 I don't like talking on the phone because I can't tell when the other person is going to say something.

IP30 After a stressful situation, I often need several hours to recover.

IP31 When people ask me for concrete examples of abstract concepts, I often can't think of any (e.g. as in answering this question).

IP32 I sometimes spend several hours a day playing out all possible outcomes of situations in my head, even if they are already in the past.

IP33 I was often frustrated and/or depressed as a child.

IP34 When people ask me questions, I often don't know what they are actually getting at and how exactly I should answer.

IP35 I can feel much more excitement when unwrapping a birthday present if I already know what's inside.

IP36 If I don't know exactly what's going to happen next, I feel nervous.

IP37 It would stress me out if I had to spontaneously take a different route to school/work.

IP38 It takes a lot of strength for me to appear "normal" to others.

IP39 I often find it difficult to determine which information is relevant to me at the moment.

IP40 I can feel emotions (e.g. joy or sadness) much more strongly when I am at home.

IP41 As a child, I often didn't understand why adults would tell me off.

IP42 The need to function in everyday life costs me a lot of energy.

IP43 When I talk about a topic that is interesting to me, I often notice that I consistently use the same choice of words.

IP44 I am completely upset if just one little thing doesn't go according to plan.

IP45 I find it much easier to be aware of my feelings when I am in a familiar environment.

IP46 I often perceive things (e.g. sounds) that other people don't seem to notice.

IP47 If several people are talking at the same time, I can't really listen to any of them.

IP48 I feel like I can't rely on anything in the world out there.

IP49 I feel a great need for stability and certainty.

IP50 During a stressful situation, I often don't realise how stressed I actually am and why exactly.

IP51 For me, getting into a romantic relationship does/would feel uncertain and/or exhausting.

IP52 If I had to learn the text for a play, I would rather memorise individual sentences and cues rather than internalising the plot as a whole.

IP53 If I have a theory about an issue and one variable turns out to be wrong, I prefer to come up with a new theory rather than update the old one.

IP54 I like doing things the same way to feel secure.

IP55_r When I'm in a room with a large group of people, I can easily follow the conversation that I want to at the time.

IP56 It can take years before I trust a person enough to feel at ease in their company.

| | |
|---|---|
| IP57 | I often experience negative feelings when I don't know what is expected of me. |
| IP58 | Often I feel overwhelmed by sounds, smells or colours. |
| IP59 | When someone is telling a story, I usually don't know where it's going to lead. |
| IP60 | It takes a long time for me to recover from everyday challenges. |
| IP61 | When something is explained to me, I need several examples to feel that I have really understood it. |
| IP62 | For me, communication is often associated with frustration and/or stress. |
| IP63 | Sometimes I feel like I just absorb all the information in a situation and can only afterwards evaluate what was important and unimportant for me. |
| IP64 | It doesn't entice me to hear my favourite band live because I like their music best the way it sounds on the CD. |
| IP65 | I often react very sensitively to the very first bite of a meal, as if my mouth had to get used to it first. |

**S7. Methodological Framework of Network Analysis**

Our item selection algorithm integrates and extends approaches from four methodological frameworks. From network psychometrics (Epskamp et al., 2018), we adopt centrality measures and community detection as structural organizers, though we specifically apply these to optimize item selection rather than merely estimating networks. From network-based scale construction (Christensen et al., 2020), we borrow the concept of using community structure to guide item selection, but diverge by prioritizing efficiency through selecting representative items rather than retaining redundant items within communities. From classical psychometrics (Furr, 2011), we incorporate item discrimination via Cohen's d and reliability assessment through Cronbach's alpha, integrating these traditional metrics with network properties. From community detection research (Fortunato, 2010), we employ community structure as a sampling frame with special consideration for singleton communities, extending these principles to psychological measurement. The resulting novel multi-strategy approach balances network structural representation (through community coverage and hub preservation) with psychometric discriminative power (through prioritizing high Cohen's d values), creating an efficient measurement tool that maintains both psychometric quality and construct coverage while minimizing redundancy.

Four complementary strategies were employed for item selection: (1) Community representation: One item with the highest Cohen's d was selected from each identified network community if it met the threshold of $d \geq 1.5$. This ensured proportional representation across the conceptual structure while maintaining discriminative power. (2) Isolated discriminators: Items with minimal network connections (degree $\leq 2$) but strong discriminative power (Cohen's $d \geq 1.6$) were selected to capture important but structurally independent constructs. (3) Central hub items: The three items with highest eigenvector centrality were included to preserve the network's structural integrity. (4) Top discriminators: The ten items with highest overall Cohen's d values were selected to maximize measurement sensitivity. Items appearing in multiple strategies were included only once, resulting in the final 18-item set. Network visualization was performed using ggraph (Pedersen, 2020) with a Fruchterman-Reingold layout to confirm adequate coverage across the identified community structure.

**S8. Discriminative Validity of the full 65-item IPPI version**

The 65-item IPPI showed strong discriminative capacity between groups. A t-test revealed significant differences, $t(352.69) = 28.17$, $p < 0.001$, with the Autism group achieving higher scores ($M = 357.18$, $SD = 46.85$) compared to the Comparison group ($M = 191.67$, $SD = 42.38$). This yielded a Cohen's $d$ of 2.85, reflecting a large effect size. ROC analysis produced an area under the curve of 0.96, indicating excellent classification performance. The optimal threshold of 288 achieved sensitivity of 0.92 and specificity of 0.90, with overall accuracy of 0.91. Classification results showed 180 of 196 autism participants and 177 of 196 comparison participants were correctly identified, totaling 35 misclassifications (16 false negatives and 19 false positives) across 392 participants. The threshold provided effective group separation with limited score distribution overlap.

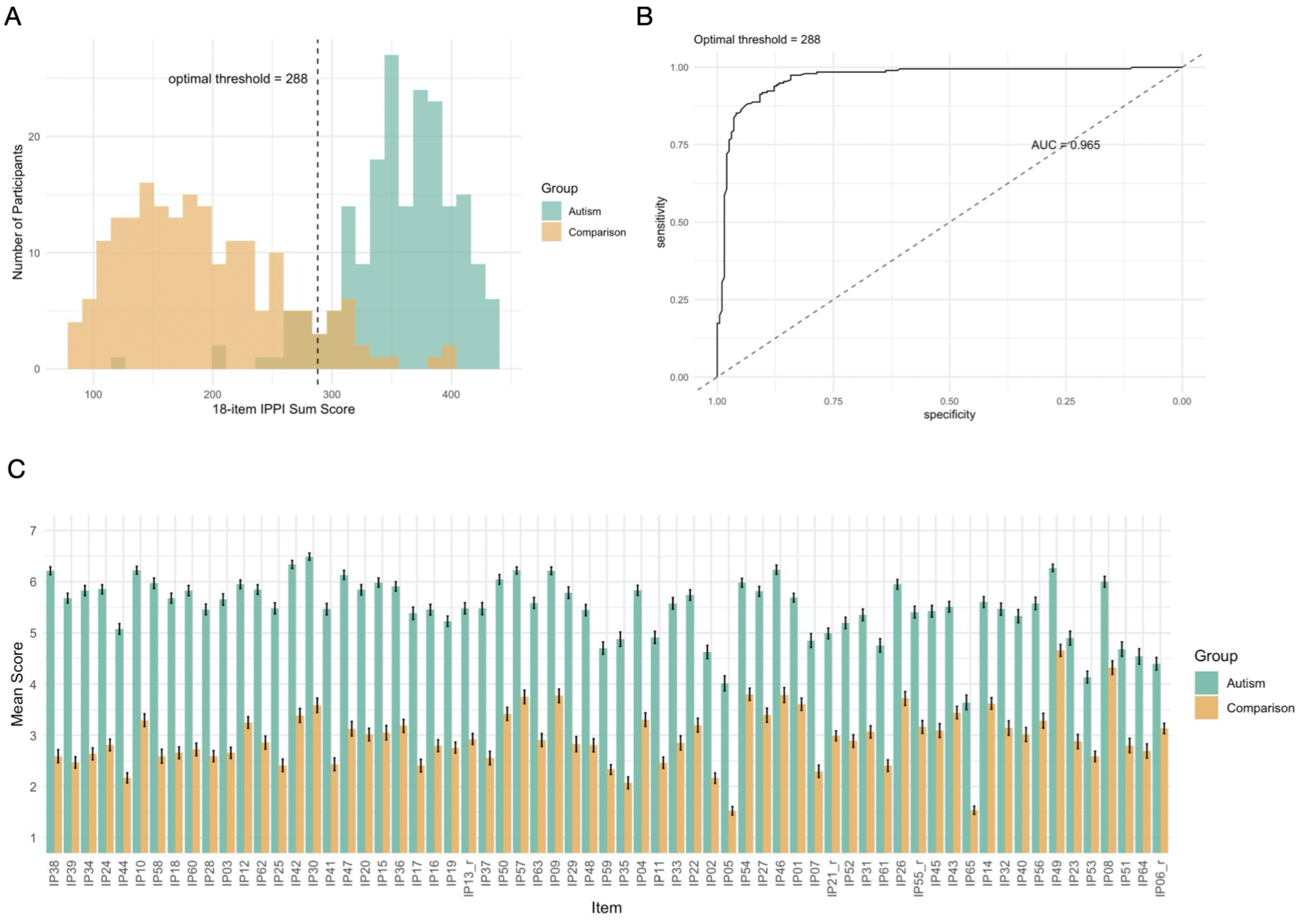

## S9. 18-items Optimized IPPI Questionnaire (German version)

| Item | Statement |
|---|---|
| IP10 | Ich habe oft das Gefühl, dass andere mich nicht verstehen. |
| IP13_r | Die Gesichtsausdrücke von Menschen sind sehr zuverlässige Informationsquellen. |
| IP17 | Ich trage sehr oft dasselbe Outfit, um mich sicher zu fühlen. |
| IP18 | Andere Menschen interpretieren meine Gesichtsausdrücke oder Gesten falsch. |
| IP24 | Ich kann nur schlecht einschätzen, wie mein Gegenüber die Dinge versteht, die ich sage. |
| IP25 | Bewegungen immer wieder zu wiederholen beruhigt mich. |
| IP28 | Wenn Dinge nicht wie erwartet eintreten, ist mein ganzer Tag ruiniert. |
| IP29 | Ich telefoniere nicht gerne, weil ich nicht weiß, wann die andere Person was sagen wird. |
| IP34 | Wenn Menschen mir Fragen stellen, weiß ich oft nicht, worauf sie eigentlich hinauswollen und wie ich genau antworten muss. |
| IP37 | Es würde mich stressen, wenn ich spontan einen anderen Weg zur Schule/Arbeit nehmen müsste. |
| IP38 | Es kostet mich sehr viel Kraft, auf andere „normal“ zu wirken. |
| IP39 | Es fällt mir oft schwer zu entscheiden, welche Informationen gerade relevant für mich sind. |
| IP44 | Es bringt mich komplett aus der Fassung, wenn nur eine Kleinigkeit nicht nach Plan läuft. |
| IP47 | Wenn mehrere Personen gleichzeitig reden, kann ich keiner davon zuhören. |
| IP50 | Während einer Stresssituation merke ich oft gar nicht, wie gestresst ich eigentlich bin und warum genau. |
| IP58 | Oft fühle ich mich von Geräuschen, Gerüchen oder Farben regelrecht überflutet. |
| IP60 | Ich brauche sehr lange, um mich von alltäglichen Herausforderungen zu erholen. |
| IP62 | Kommunikation ist für mich oft mit Frust und/oder Stress verbunden. |

**S10. Description of Factor Structure**

*Factor and Item descriptions represent hypothetical conceptual relations to predictive processing theory.*

**Factor 1: Prediction Integration and Interpretation**
This factor captures challenges in integrating sensory/social inputs with existing predictive models and interpreting prediction errors across domains

| Item | Statement | Predictive Processing Relevance |
|---|---|---|
| IP10 | I often have the feeling that others don't understand me. | Reflects prediction errors arising from divergent generative models between communication partners, where mutual predictability is compromised due to differences in predictive frameworks. |
| IP13_r | People's facial expressions are very reliable sources of information. | Reflects lower precision weighting assigned to facial expressions as predictive cues, recognizing that these signals may be ambiguous, context-dependent, or inconsistently informative rather than universally reliable. |
| IP17 | I like to wear the same outfit over and over again to make me feel at ease. | Indicates preference for sensory predictability and minimizing prediction errors through consistency in tactile and visual sensory input. |
| IP18 | Other people misinterpret my facial expressions or gestures. | Reflects prediction errors arising from divergent generative models for nonverbal communication, where different expression and interpretation frameworks between interaction partners lead to systematic misalignment in social signaling. |
| IP24 | I have a hard time guessing how another person might understand the things I say. | Reflects challenges in predicting others' interpretive frameworks when different generative models for communication create uncertainty about how messages will be received. |
| IP34 | When people ask me questions, I often don't know what they are actually getting at and how exactly I should answer. | Reflects prediction errors when inferring unstated intentions behind questions, indicating mismatches between question-framing and interpretation frameworks. |
| IP38 | It takes a lot of strength for me to appear "normal" to others. | Represents the effortful process of overriding natural responses to match social expectations, highlighting the cognitive load of maintaining non-default predictive models. |
| IP39 | I often find it difficult to determine which information is relevant to me at the moment. | Captures precision-weighting challenges in predictive processing—difficulties assigning appropriate relevance to incoming information. |
| IP47 | If several people are talking at the same time, I can't really listen to any of them. | Reflects impairments in predictive filtering of auditory information, showing challenges in using top-down predictions to isolate relevant speech. |
| IP50 | During a stressful situation, I often don't realise how stressed I actually am and why exactly. | Indicates reduced interoceptive prediction accuracy—difficulty integrating bodily signals into conscious awareness through predictive models. |
| IP58 | Often I feel overwhelmed by sounds, | Demonstrates sensory prediction errors—excessive |

| | | |
|---|---|---|
| | smells or colours. | precision assigned to bottom-up sensory inputs without sufficient dampening by top-down predictions. |
| IP62 | For me, communication is often associated with frustration and/or stress. | Represents the emotional consequences of persistent prediction errors in social communication, showing how failed integration leads to negative affect. |

**Factor 2: Prediction Error Sensitivity and Stability Needs**
This factor reflects the need for environmental predictability, difficulties with unexpected changes, and distress responses to prediction violations

| Item | Statement | Predictive Processing Relevance |
|---|---|---|
| IP25 | It is calming to repeat movements over and over again. | Reflects how repetitive behaviors help maintain a stable, predictable sensory-motor environment with minimal prediction errors, creating a calming effect through consistent sensory feedback. |
| IP28 | It ruins my whole day, if things don't happen as expected. | Captures the significant emotional impact of prediction violations, showing how unexpected events can trigger widespread prediction errors across domains, leading to prolonged distress. |
| IP29 | I don't like talking on the phone because I can't tell when the other person is going to say something. | Demonstrates how unpredictable conversational timing creates prediction uncertainty, highlighting the importance of temporal predictability in communication. |
| IP37 | It would stress me out if I had to spontaneously take a different route to school/work. | Illustrates how disruptions to established environmental predictions (route navigation) generate stress, reflecting the preference for maintaining established predictive models. |
| IP44 | I am completely upset if just one little thing doesn't go according to plan. | Shows the disproportionate impact of even minor prediction errors, highlighting the precision with which predictions are monitored and the sensitivity to deviations. |
| IP60 | It takes a long time for me to recover from everyday challenges. | Reflects the extended allostatic processing required to integrate prediction errors and restore predictive equilibrium after encountering unexpected situations. |

**S11. Convergent Validity in Sample 1**

*Scatterplots illustrating correlations between IPPI, AQ and BAP-Q*

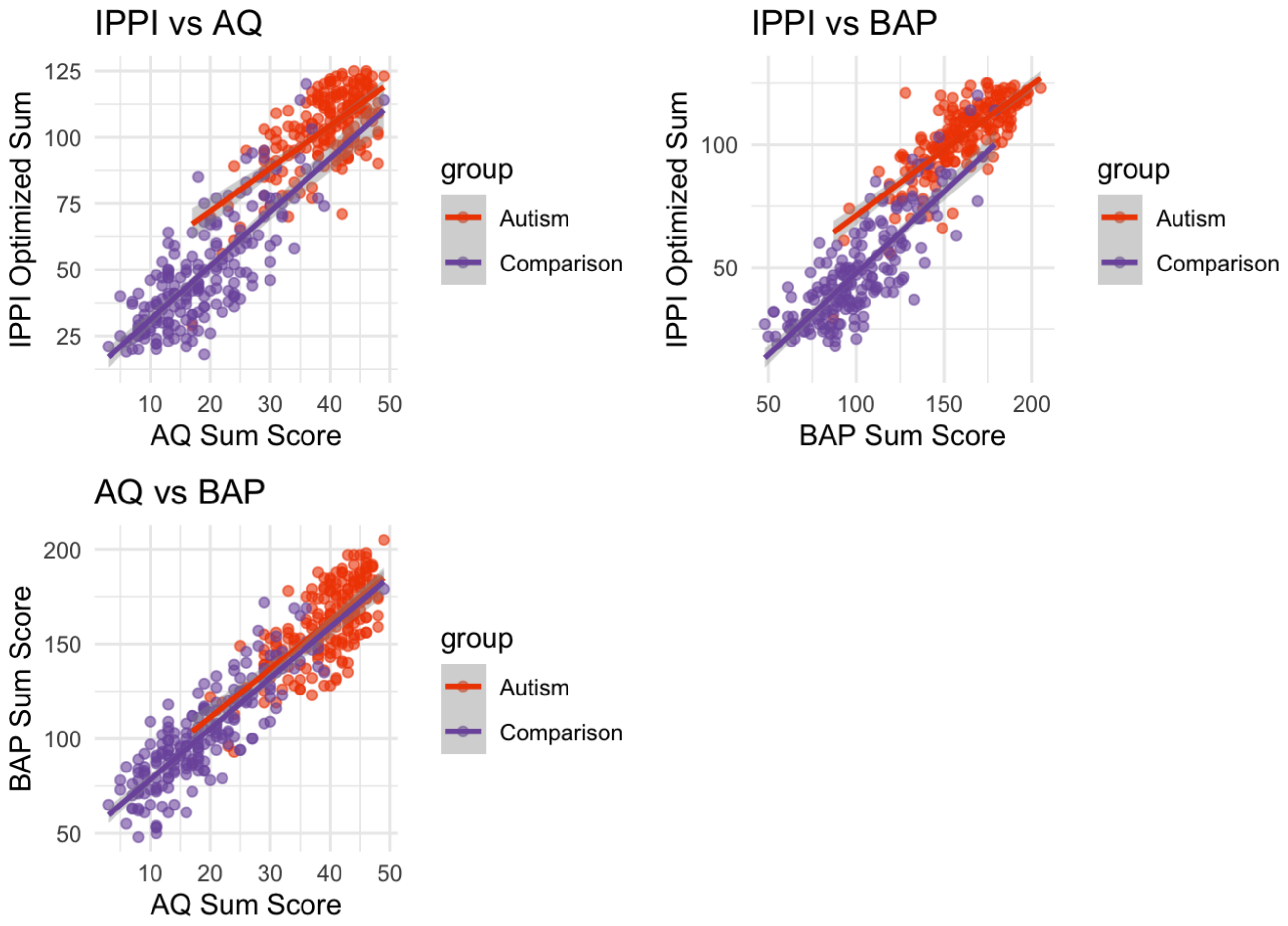


**References**

Austin, P. C. (2011). An introduction to propensity score methods for reducing the effects of confounding in observational studies. *Multivariate Behavioral Research*, *46*(3), 399-424. https://doi.org/10.1080/00273171.2011.568786

Christensen, A. P., Golino, H., & Silvia, P. J. (2020). A psychometric network perspective on the validity and validation of personality trait questionnaires. *European Journal of Personality*, *34*(6), 1095-1108. https://doi.org/10.1002/per.2265

Epskamp, S., Borsboom, D., & Fried, E. I. (2018). Estimating psychological networks and their accuracy: A tutorial paper. *Behavior Research Methods*, *50*(1), 195-212. https://doi.org/10.3758/s13428-017-0862-1

Fortunato, S. (2010). Community detection in graphs. *Physics Reports*, *486*(3-5), 75-174. https://doi.org/10.1016/j.physrep.2009.11.002

Furr, R. M. (2011). *Scale construction and psychometrics for social and personality psychology*. SAGE Publications.

Ho, D., Imai, K., King, G., & Stuart, E. A. (2011). MatchIt: Nonparametric preprocessing for parametric causal inference. *Journal of Statistical Software*, *42*, 1-28. https://doi.org/10.18637/jss.v042.i08

Pedersen, T. L. (2020). ggraph: An implementation of grammar of graphics for graphs and networks. R package version 2.0.5. https://CRAN.R-project.org/package=ggraph

Rosenbaum, P. R., & Rubin, D. B. (1983). The central role of the propensity score in observational studies for causal effects. *Biometrika*, *70*(1), 41-55. https://doi.org/10.1093/biomet/70.1.41
